# O058. Cluster headache with accompanying migraine-like features

**DOI:** 10.1186/1129-2377-16-S1-A93

**Published:** 2015-09-28

**Authors:** Arens Taga, Marco Russo, Gian Camillo Manzoni, Paola Torelli

**Affiliations:** Parma Headache Centre, Department of Clinical and Experimental Medicine, University of Parma, Parma, Italy

## Background

There are limited literature data on migraine-like accompanying features (MLF) in patients with cluster headache (CH). These symptoms are frequently reported by patients[1–3] and may delay CH diagnosis[4].

The aim of the present study was to investigate the occurrence of migraine symptoms in an Italian case series of CH patients, and to determine whether these features influence the clinical phenotype of CH.

## Methods

A retrospective cohort study was performed in all consecutive patients referred to the Parma Headache Centre between 1975 and 2013 affected by CH, diagnosed by our team of trained neurologists; the cases were subsequently reviewed applying the ICHD3-beta criteria (785 patients, 569 men and 216 women)[5]. We then identified those patients who reported at least one of the migraine accompanying symptoms (i.e., nausea or vomiting or photophobia and phonophobia); the remaining patients were considered as CH cases without MLF.

## Results

We identified 362 patients (250 men and 112 women) reporting MLF and 423 patients (319 men and 104 women) without MLF, with a significantly lower M:F ratio in the first group; furthermore these patients had a lower mean age of headache onset (27.2±12.1 yrs vs 32.3±14.3 yrs).

There were no significant disproportions between the two groups regarding CH subtype diagnosis (i.e., episodic and chronic diagnosis), the presence of CH in first-degree relatives and the occurrence of comorbid migraine or of a prior head injury. Smoking habits were significantly less frequent among patients with MLF while alcohol intake was similar.

We found significant differences in CH time course: attack duration was longer among patients with MLF (89.0±88.9 minutes vs 76.3±68.3 minutes), while there was a lower number of attacks per day (1.5±0.9 vs 2.0±1.6) only among women. We did not find differences in cluster bouts duration and frequency.

Patients with MLF had a higher number of cranial autonomic symptoms (3.2±1.5 vs 2.8±1.6), with a significantly higher proportion of lacrimation, conjunctival injection, rhinorrhoea, facial sweating and miosis occurring in men only; ptosis was more frequent in both men and women with MLF. Other clinical features such as pain side, pain intensity and restlessness were similarly distributed.

## Conclusions

Our study confirms the high proportion of CH patients with MLF which is reported in the literature[1–3]. The presence of MLF seems to relate to some peculiar demographic and clinical characteristics of CH sufferers. Whether these features influence the response to therapy remains to be determined.

Written informed consent to publication was obtained from the patient(s).
